# Prevalence of Antiphospholipid Antibodies and Association With Incident Cardiovascular Events

**DOI:** 10.1001/jamanetworkopen.2023.6530

**Published:** 2023-04-04

**Authors:** Yu Zuo, Sherwin Navaz, Wenying Liang, Chun Li, Colby R. Ayers, Christine E. Rysenga, Alyssa Harbaugh, Gary L. Norman, E. Blair Solow, Bonnie Bermas, Oludamilola Akinmolayemi, Anand Rohatgi, David R. Karp, Jason S. Knight, James A. de Lemos

**Affiliations:** 1 Division of Rheumatology, Department of Internal Medicine, University of Michigan, Ann Arbor; 2 Division of Cardiology, Department of Internal Medicine, University of Texas Southwestern Medical Center, Dallas; 3 Division of Rheumatic Disease, Department of Internal Medicine, University of Texas Southwestern Medical Center, Dallas; 4 Department of Rheumatology and Immunology, Peking University People's Hospital, Beijing, China; 5 Headquarters & Technology Center Autoimmunity, Werfen, San Diego, California; 6 Department of Internal Medicine, New York-Presbyterian/Columbia University Irving Medical Center, New York

## Abstract

**Importance:**

The prevalence of antiphospholipid antibodies (aPL) and their association with future atherosclerotic cardiovascular disease (ASCVD) risk has yet to be thoroughly investigated.

**Objective:**

To determine the association between measurements of aPL at a single time point and ASCVD risk in a diverse population.

**Design, Setting, and Participants:**

This cohort study measured 8 aPL (anticardiolipin [aCL] IgG/IgM/IgA, anti–beta-2 glycoprotein I [aβ2GPI] IgG/IgM/IgA, and antiphosphatidylserine/prothrombin [aPS/PT] IgG/IgM) by solid-phase assays in plasma from participants of the Dallas Heart Study (DHS) phase 2, a multiethnic, population-based cohort study. Blood samples were collected between 2007 and 2009. The median follow-up was 8 years. Statistical analysis was performed from April 2022 to January 2023.

**Main Outcomes and Measures:**

Associations of aPL with future ASCVD events (defined as first nonfatal myocardial infarction, first nonfatal stroke, coronary revascularization, or death from cardiovascular cause) were assessed by Cox proportional hazards models, adjusting for known risk factors, medications, and multiple comparisons.

**Results:**

Among the 2427 participants (mean [SD] age, 50.6 [10.3] years; 1399 [57.6%] female; 1244 [51.3%] Black, 339 [14.0%] Hispanic, and 796 [32.8%] White), the prevalence of any positive aPL tested at a single time point was 14.5% (353 of 2427), with approximately one-third of those detected at a moderate or high titer; aCL IgM had the highest prevalence (156 individuals [6.4%]), followed by aPS/PT IgM (88 [3.4%]), aβ2GPI IgM (63 [2.6%]), and aβ2GPI IgA (62 [2.5%]). The IgA of aCL (adjusted hazard ratio [HR], 4.92; 95% CI, 1.52-15.98) and aβ2GPI (HR, 2.91; 95% CI, 1.32-6.41) were independently associated with future ASCVD events. The risk further increased when applying a positivity threshold of at least 40 units (aCL IgA: HR, 9.01 [95% CI, 2.73-29.72]; aβ2GPI IgA: HR, 4.09 [95% CI, 1.45-11.54]). Levels of aβ2GPI IgA negatively correlated with cholesterol efflux capacity (*r* = −0.055; *P* = .009) and positively correlated with circulating oxidized LDL (*r* = 0.055; *P* = .007). aβ2GPI IgA–positive plasma was associated with an activated endothelial cell phenotype as evidenced by increased surface expression of surface E-selectin, intercellular adhesion molecule-1, and vascular cell adhesion molecule-1.

**Conclusions and Relevance:**

In this population-based cohort study, aPL detectable by solid-phase assays were present in a substantial proportion of adults; positive aCL IgA and aβ2GPI IgA at a single time point were independently associated with future ASCVD events. Longitudinal studies with serial aPL measurements are needed to further explore these findings.

## Key Points

**Question:**

At a single time point without confirmatory testing, what is the prevalence of antiphospholipid antibodies (aPL), and are they associated with future atherosclerotic cardiovascular disease (ASCVD) risk?

**Findings:**

In this population-based cohort study including 2427 participants, the prevalence of any aPL tested by solid-phase assays at a single time point was 14.5%, with approximately one-third of those detected at a moderate or high titer. The IgA isotypes of anticardiolipin and anti–beta-2 glycoprotein I were associated with future ASCVD events.

**Meaning:**

These results suggest that some aPL are associated with increased ASCVD risk in the general population; longitudinal studies with serial laboratory assessments will be needed to further explore these associations.

## Teaser

This cohort study examines the association between measurements of antiphospholipid antibodies (aPL) at a single time point and atherosclerotic cardiovascular disease risk in a diverse population.

## Introduction

Individuals with autoimmune and inflammatory diseases have greater risk of cardiovascular events than expected based on traditional atherosclerotic cardiovascular disease (ASCVD) risk factors.^1,2^ Mechanisms proposed to explain this risk include inflammation-mediated disruption of vascular integrity and activation of platelets and coagulation pathways resulting in cardiovascular tissue remodeling. However, the role of autoantibodies specifically in the natural history of ASCVD remains unclear.^2^ A recent study suggested autoantibodies are present in as many as 18% to 32% of individuals,^3^ percentages that may further increase following the COVID-19 pandemic.^4,5^ Although most of these individuals are unlikely to be diagnosed with a named autoimmune disease, they may be at higher risk for ASCVD morbidity than is currently recognized. Defining the association of autoimmunity with ASCVD risk is a timely imperative.

Antiphospholipid antibodies (aPL) are an important but heterogeneous group of autoantibodies that activate endothelial cells, platelets, and neutrophils via interactions with cell-associated phospholipids and phospholipid-binding proteins such as beta-2-glycoprotein I (β2GPI) and prothrombin.^6,7,8,9,10,11^ Antiphospholipid syndrome (APS) is an acquired thromboinflammatory disease characterized by arterial, venous, and microvascular thrombotic events and obstetric complications in the presence of persistently circulating aPL.^12^ In patients with APS, arterial occlusions leading to stroke and myocardial infarction are among the most common causes of morbidity and mortality.^13^ Current APS classification criteria require evidence of persistently abnormal test results (over at least 12 weeks) by solid-phase assays (anticardiolipin [aCL] IgG/IgM, anti-β2GPI [aβ2GPI] IgG/IgM), or by the lupus anticoagulant functional assay.^14,15^

Several noncriteria aPL, such as antiphosphatidylserine/prothrombin (aPS/PT) IgG/IgM and the IgA isotypes of aCL and aβ2GPI are not included in the current APS classification criteria,^14^ but at times still identify individuals with APS-related clinical manifestations.^6,15^ aPS/PT recognizes prothrombin complexes with anionic phosphatidylserine and associates closely with positive lupus anticoagulant testing.^7^ A systematic review of 10 retrospective studies found a significant association between aPS/PT positivity and thromboembolic events.^8^ The IgA isotypes of aCL and aβ2GPI have also been shown to associate with features of APS such as thrombosis and pregnancy loss, perhaps especially among patients with systemic lupus erythematosus (SLE).^9,10,11,16^

Cross-sectional studies have shown that aPL are acutely present in up to 17.4% of patients with cardiovascular events such as stroke and transient ischemic attack.^17,18^ Small cohort studies have suggested that positive aPL testing may be present in 1% to 12% of seemingly healthy individuals.^19,20^ Inconsistent aPL testing methods and short follow-up times have limited previous studies. Furthermore, as disparities in health care are rightfully receiving increasing attention, the impact of sex, race, and ethnicity on aPL prevalence and associated ASCVD risk remains to be elucidated. This study aimed to determine the association between aPL and future ASCVD events in a large, racially and ethnically diverse population and to suggest potential mechanisms underlying a pathogenic role that some aPL may play in ASCVD events. We also sought to investigate the association of sex, race, and ethnicity with aPL prevalence.

## Methods

### Study Population

2427 participants from Dallas Heart Study phase 2 (DHS2) who were free of ASCVD events at the time of blood collection were included.^21^ No participants had self-reported autoimmune diseases requiring immunosuppressive medications. Race and ethnicity were self-reported by participants. To assess ASCVD risk in a diverse population, race and ethnicity data were collected for the Dallas Heart Study (DHS). Written informed consent was obtained from all DHS participants. The University of Texas Southwestern institutional review board approved the study protocol. University of Michigan’s institutional review exempted this substudy. We followed the Strengthening the Reporting of Observational Studies in Epidemiology (STROBE) reporting guideline. Additional study population details are in the eMethods of Supplement 1.

### Quantification of aPL

aPL were quantified from plasma using Quanta Lite kits (Werfen North America).^22^ See eMethods of Supplement 1 for additional details.

### Determination of ASCVD Events

ASCVD events, defined as first nonfatal myocardial infarction, first nonfatal stroke, coronary or peripheral artery revascularization, or cardiovascular death, were systematically ascertained and adjudicated as previously described.^23^

### Quantification of Antinuclear Antibodies

Antinuclear antibodies (ANA) were quantified in plasma collected during DHS1 using Quanta Lite ANA (Inova Diagnostics).^24^ ANA was not quantified at the DHS2 visit.

### Cholesterol Efflux Capacity

The detailed methodology has been previously described for cholesterol efflux capacity (CEC).^25,26^ See eMethods of Supplement 1 for additional details.

### Circulating Neutrophil Extracellular Trap Remnants

We identified 25 DHS participants who were aβ2GPI IgA–positive and matched each with 3 aβ2GPI IgA–negative control participants based on age, sex, and race and ethnicity. Two markers of neutrophil extracellular trap (NET) remnants were assessed, myeloperoxidase-DNA complexes and citrullinated histone H3.^27^ Myeloperoxidase-DNA complexes were quantified as has been previously described (eMethods in Supplement 1).^28^ Citrullinated histone H3 was quantified using the Citrullinated Histone H3 Enzyme-Linked Immunoassay (ELISA) Kit (Cayman, 501620) according to the manufacturer’s instructions.

### In-Cell ELISA

Human coronary artery endothelial cell activation was assessed by an in-cell ELISA, which measured surface expression of E-selectin, intercellular adhesion molecule-1 (ICAM-1), and vascular cell adhesion molecule-1 (VCAM-1).^29^ See eMethods of Supplement 1 for additional details.

### Statistical Analysis

aPL were analyzed as categorical variables (based on both the manufacturer’s and the moderate- or high-titer [≥40 units] threshold). Sex and ethnicity variation were compared using χ^2^ or Jonckheere-Terpstra trend test. Association with ANA was determined using the χ^2^ test. Spearman correlations were performed to assess the association between CEC (continuous), oxidized LDL (continuous), and aPL levels (continuous). Associations of various aPL with future ASCVD events were assessed by Cox proportional hazards models with and without adjustment for known cardiovascular disease risk factors including age, sex, race ethnicity, body mass index (BMI), smoking history, systolic blood pressure, diabetes, total cholesterol, high-density lipoprotein cholesterol (HDL), and kidney function (based on glomerular filtration rate [GFR] estimated by MDRD method) and medication use including aspirin, statins, and any antihypertensives. Additional analyses were performed to test for interactions between race and ethnicity, aPL subtypes, and ASCVD events. Sensitivity analyses were performed replacing total cholesterol and HDL with LDL and triglycerides. Levels of circulating NET remnants and surface expression of endothelial adhesion molecules were compared between groups using the Mann-Whitney test. Two-sided *P* < .05 was considered statistically significant. Adjustment for multiple testing was done using the modified Benjamini-Hochberg Step-up method. All statistical analyses were performed using SAS version 9.4 (SAS Institute) from April 2022 to January 2023 (eMethods in Supplement 1).

## Results

Among the 2427 participants in DHS2 (blood samples collected between 2007 and 2009) included in this study, 1399 (57.6%) were female, 1244 (51.3%) were Black, 339 (14.0%) were Hispanic, and 796 (32.8%) were White; the mean (SD) age at the time of sampling was 50.6 (10.3) years. All were free of CVD and had no self-reported autoimmune disease requiring immunosuppressive medications at the time of the DHS2 visit. Characteristics of the study population are shown in Table 1.

**Table 1.** Demographic and Clinical Characteristics of Dallas Heart Study Participants

Characteristics	Participants, No. (%)			*P* value^b^
	All DHS participants (n = 2427)	Any aPL positive^a^ (n = 353)	All aPL negative (n = 2074)	
Demographics				
Age, mean (SD), y	50.6 (10.3)	53.6 (10.3)	50.4 (10.3)	NA
Sex				
Female	1399 (57.6)	222 (62.9)	1177 (56.8)	.03
Male	1028 (42.4)	131 (37.1)	897 (43.2)	.19
Race and ethnicity				
Black	1244 (51.3)	198 (56.1)	1046 (50.4)	.05
Hispanic	339 (14.0)	51 (14.4)	288 (13.8)	.78
White	796 (32.8)	102 (28.9)	694 (33.5)	.09
Other^c^	48 (2.0)	2 (0.6)	46 (2.2)	.05
Other cardiovascular risks				
BMI, mean (SD)	31.2 (7.5)	32.0 (7.6)	31.1 (7.4)	.02
BP, mean (SD), mm Hg				
Systolic	133 (20)	135 (22)	132 (20)	.03
Diastolic	81 (9)	81 (10)	81 (9)	.33
HbA_1c_, mean (SD), %	5.7 (1.1)	5.8 (1.2)	5.8 (1.1)	.52
LDL cholesterol, mean (SD), mg/dL	115 (36)	112 (35)	116 (36)	.05
HDL cholesterol, mean (SD), mg/dL	52 (15)	52 (15)	52 (15)	.91
Triglycerides, mean (SD), mg/dL	125 (86)	126 (82)	125 (87)	.18
Current smoker	532 (22.0)	77 (21.8)	455 (22.0)	.97

Abbreviations: aPL, antiphospholipid antibodies; BMI, body mass index (calculated as weight in kilograms divided by height in meters squared); BP, blood pressure; DHS, Dallas Heart Study; HbA_1C_, hemoglobin A_1C_; HDL, high-density lipoprotein; LDL, low-density lipoprotein.

SI conversion factors: To convert cholesterol to millimoles per liter, multiply by 0.0259; triglycerides to millimoles per liter, multiply by 0.0113.

^a^ Based on the manufacturer’s cutoff.

^b^ Compared demographic and clinical characteristics between participants who were aPL-positive and participants who were aPL-negative.

^c^ Included Asian or Pacific Islander, American Indian or Alaska Native, and other self-reported race and ethnicity by Dallas Heart Study participants.

### Prevalence of aPL Detected by Solid-Phase Assays

There were 353 individuals who tested positive for at least 1 aPL based on the manufacturer’s threshold, representing 14.5% of the cohort (Table 2). The mean (SD) age for aPL-positive participants was 53.6 (10.3) years, as compared with 50.4 (10.3) years for aPL-negative participants. Among the aPL tested, aCL IgM had the highest prevalence (156 participants [6.4%]), followed by aPS/PT IgM (88 participants [3.4%]), aβ2GPI IgM (63 individuals [2.6%]), and aβ2GPI IgA (62 participants [2.5%]); 17 participants (0.7%) were positive for 3 or more. Among the 353 participants with positive aPL, 65 participants (18.4%) had positive testing for at least 1 IgG isotype. 153 participants (6.3%) had at least 1 aPL at moderate or high titer, defined as at least 40 units based on the typical convention of APS field (Table 2).

**Table 2.** Prevalence of Antiphospholipid Antibodies in Dallas Heart Study Population (N = 2427)

aPL	No. positive (%)	
	Manufacturer’s threshold^a^	Titer ≥40 units
aCL IgG^b^	26 (1.0)	7 (0.3)
aCL IgM^b^	156 (6.4)	36 (1.5)
aCL IgA	11 (0.5)	6 (0.3)
aβ2GPI IgG^b^	21 (0.9)	10 (0.4)
aβ2GPI IgM^b^	63 (2.6)	26 (1.0)
aβ2GPI IgA	62 (2.5)	29 (1.2)
aPS/PT IgG	18 (0.7)	11 (0.5)
aPS/PT IgM	88 (3.4)	48 (2.0)
Any positive	353 (14.5)	153 (6.3)
Three positive aPL	17 (0.7)	2 (0.08)

Abbreviations: aβ2GPI, anti–beta-2 glycoprotein I; aCL, anticardiolipin; aPL, antiphospholipid antibodies; APS, antiphospholipid syndrome; aPS/PT, anti-phosphatidylserine/prothrombin.

^a^ Manufacturer’s thresholds: aCL IgG/M/A = 20 GPL/MPL/APL; aβ2GPI IgG/M/A = 20 SGU/SMU/SAU; aPS/PT IgG/M = 30 arbitrary units.

^b^ Antiphospholipid antibodies that are part of current APS classification criteria. Others are characterized as noncriteria aPL.

### Prevalence of aPL by Sex, Race, and Ethnicity

Positive aCL IgM and aPS/PT IgM were observed more frequently in female participants than in male participants (eTable 1 in Supplement 1). However, these differences disappeared when applying the more stringent threshold of at least 40 units (eTable 2 in Supplement 1). Although Hispanic participants had the highest frequency of aβ2GPI IgA, no significant differences were found in the frequency of aPL (any test positive) between Black, Hispanic, and White participants (Table 3). Similar patterns were seen when applying the more stringent threshold (eTable 3 in Supplement 1).

**Table 3.** Racial and Ethnic Variation of Antiphospholipid Antibodies in Dallas Heart Study (N = 2427)

aPL	No. positive (% manufacturer’s threshold)				Adjusted *P* value^b^
	Black (n = 1244)	Hispanic (n = 339)	White (n = 796)	Other^a^ (n = 48)	
aCL IgG	15 (1.2)	1 (0.3)	10 (1.3)	0	.54
aCL IgM	84 (6.8)	16 (4.7)	55 (6.9)	1 (2.0)	.54
aCL IgA	8 (0.6)	1 (0.3)	2 (0.3)	0	.61
aβ2GPI IgG	15 (1.2)	2 (0.6)	4 (0.5)	0	.54
aβ2GPI IgM	27 (2.2)	5 (1.5)	30 (3.8)	1 (2.0)	.23
aβ2GPI IgA	37 (3.0)	22 (6.5)	2 (0.3)	1 (2.0)	<.001
aPS/PT IgG	12 (1.0)	3 (0.9)	3 (0.4)	0	.54
aPS/PT IgM	48 (3.9)	11 (3.2)	23 (2.9)	0	.54
Any positive	198 (15.9)	51 (15)	102 (12.8)	2 (4.0)	.23
Three positive aPL	11 (0.9)	2 (0.6)	4 (0.5)	0	.69

Abbreviations: aβ2GPI, anti–beta-2 glycoprotein I; aCL, anticardiolipin; aPL, antiphospholipid antibodies; aPS/PT, anti-phosphatidylserine/prothrombin.

^a^ Included Asian or Pacific Islander, American Indian or Alaska Native, and other self-reported race and ethnicity by Dallas Heart Study participants.

^b^ Adjusted for multiple comparison by modified Benjamini-Hochberg step-up method.

### Association Between Positive ANA and aPL

Among 2427 participants, 2155 had ANA assessed at the DHS1 study visit (1999 to 2001); 103 out of 2155 (4.8%) were positive, in line with the reported population prevalence of ANA.^24,30,31,32^ Positive ANA (equivalent to at least 1:160) was significantly associated with moderate- or high-titer positive aβ2GPI IgA (*P* = .02), aPS/PT IgG (*P* = .001), aPS/PT IgM (*P* = .003), and any aPL (*P* = .001) (eTable 4 in Supplement 1).

### Association With Future ASCVD Events

**Figure 1.**
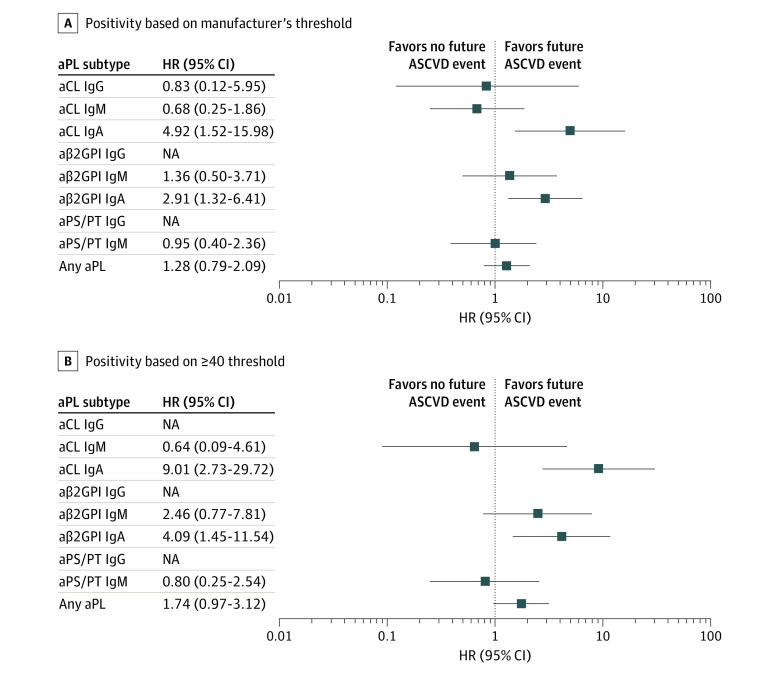
Association of aPL Subtypes With Future Atherosclerotic Cardiovascular Disease (ASCVD) Events Association between positive aPL based on either manufacturer’s threshold (A) or at least 40 units threshold (B) and future ASCVD events were assessed in Cox proportional hazard models adjusted for known cardiovascular risks including age, ethnicity, smoking history, hypertension, diabetes, and lipid profiles. aCL indicates anticardiolipin; aPL, antiphospholipid antibodies; aPS/PT, antiphosphatidylserine/prothrombin; aβ2GPI, anti–beta-2 glycoprotein I; HR, hazard ratio; NA, not applicable (HR not calculable due to 0 events in aPL group).

Over a median follow-up period of 8 years, ASCVD events occurred in 125 participants, including first nonfatal MI in 79 participants, first non-fatal stroke in 74, percutaneous coronary intervention in 90, coronary-artery bypass grafting in 41, and cardiovascular death in 52. After adjusting for age, sex, race, ethnicity, BMI, smoking history, systolic blood pressure, diabetes, total cholesterol, HDL, eGFR, medications, and multiple comparisons, positive testing for aCL IgA and aβ2GPI IgA were each significantly associated with future ASCVD events. Based on the manufacturer’s threshold, the adjusted hazard ratio (HR) for aCL IgA was 4.92 (95% CI, 1.52-15.98). The adjusted HR for aβ2GPI IgA was 2.91 (95% CI, 1.32-6.41). Using the more stringent threshold of at least 40 units, the adjusted HR for aCL IgA increased to 9.01 (95% CI, 2.73-29.72), and the adjusted HR for aβ2GPI IgA increased to 4.09 (95% CI, 1.45-11.54) (Figure 1). To further improve overall coverage probability, we also assessed and reported 95% simultaneous CI, which did not change the findings (eFigure 1 in Supplement 1). The results from the unadjusted model are presented in eTable 5 in Supplement 1. Replacing total cholesterol and HDL with LDL and triglycerides in the model did not change the overall results. Furthermore, interaction analyses between aPL subtypes and race and ethnicity were assessed in the models, and no significant interactions were found.

### Association With CEC and Oxidized Low-Density Lipoprotein

aβ2GPI IgA was negatively correlated with CEC (*r* = −0.055; *P* = .009), and was positively correlated with circulating oxidized LDL (*r* = 0.055; *P* = .007). No significant correlations were found for aCL IgA (CEC *r* = −0.011; *P* = .59; oxidized LDL *r* = .001; *P* = .98).

### aβ2GPI IgA and NET Remnants

There were 25 DHS participants selected who were aβ2GPI IgA–positive and 75 age- and sex-matched participants who were aβ2GPI IgA–negative were selected. Two markers of circulating NET remnants, myeloperoxidase-DNA complexes and citrullinated histone H3, were evaluated in this cohort. No significant differences in circulating NET remnants were found between the groups (eFigure 2 in Supplement 1).

### aβ2GPI IgA–Positive Plasma on Human Coronary Artery Endothelial Cells

**Figure 2.**
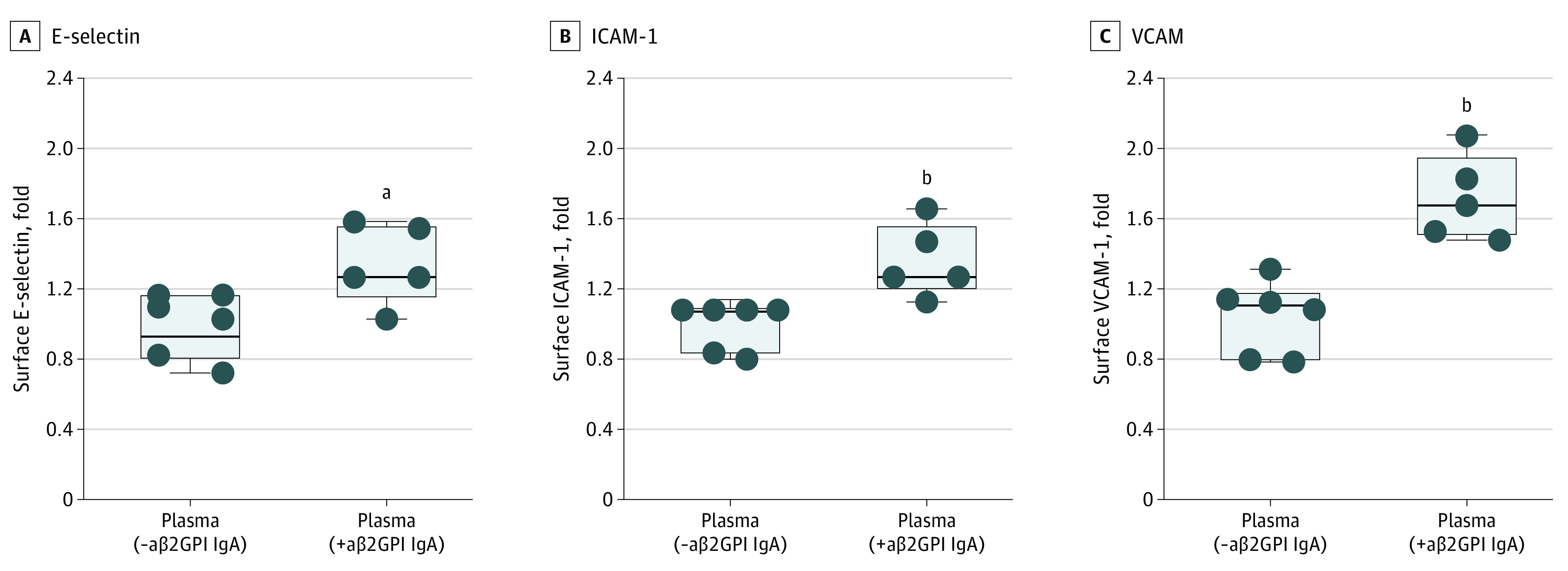
Activation of Human Coronary Artery Endothelial Cells (HCEC) by aβ2GPI IgA–Positive or aβ2GPI IgA–Negative Plasma In-cell enzyme-linked immunoassay for HCEC was performed (see eFigure 3 in Supplement 1). HCEC were cultured for 6 hours with plasma from DHS2 participants who were either aβ2GPI-positive or aβ2GPI-negative. Cells were then fixed, and surface expression of E-selectin (B), ICAM-1 (C), or VCAM-1 (D) was quantified. Median, upper, and lower quartile, and range are indicated with Box and Whisker plot. Groups were analyzed by Mann-Whitney test. aβ2GPI indicates anti–beta-2 glycoprotein I; ICAM-1, intercellular adhesion molecule-1; VCAM-1, vascular cell adhesion molecule-1. ^a^*P* < .05. ^b^*P* < .01.

Plasma samples of aβ2GPI IgA–positive individuals (and controls from the aforementioned matched cohort) with sufficient remaining plasma volume were added to early-passage human coronary artery endothelial cells. The expression of cell adhesion molecules was determined after 6 hours via an in-cell ELISA platform.^29^ As compared with aβ2GPI IgA–negative plasma, aβ2GPI IgA–positive plasma was associated with an activated endothelial cell phenotype as evidenced by increased surface expression of surface E-selectin (Figure 2A), ICAM-1 (Figure 2B), and VCAM-1 (Figure 2C).

## Discussion

In our representative, racially and ethnically diverse population-based cohort, at least 1 type of aPL was detected by solid-phase assays at a single time point in 14.5% of individuals, with approximately one-third of the aPL present at moderate or high titer. The majority of positives were associated with 4 antibodies: aCL IgM, aPS/PT IgM, aβ2GPI IgM, and aβ2GPI IgA. Little variation based on race and ethnicity was noted, contrasting with previous assertions that aPL IgG and IgM may be less common in Black individuals.^33,34^

Our data suggest that the presence of certain aPL, particularly the IgA isotypes of aCL and aβ2GPI, even when measured at a single time point, are associated with future ASCVD events. We can speculate that aβ2GPI IgA might increase ASCVD risk by impairing HDL function and/or increasing proatherogenic circulating oxidized LDL. Although NETs have previously been shown to impair HDL function,^35^ that is unlikely to be the mechanism here given no differences in 2 assays for NETs between individuals who were aβ2GPI IgA–positive and aβ2GPI IgA–negative. Beyond HDL, dysfunctional endothelial cells are also important in orchestrating atherosclerotic plaque formation.^36,37^ Here, our data preliminarily suggest that exposure to aβ2GPI IgA may shift coronary endothelial cells toward a pro-adhesive and atherogenic phenotype, although definitive evidence of a causal relationship will require further experimentation.

The population prevalence of aPL, as detected by solid-phase assays, has yet to be definitively elucidated in diverse populations.^17^ Available aPL epidemiology is limited by variability in study design, definitions of positive aPL, and the absence of important noncriteria aPL.^17^ Larger population-based studies, such as the Honolulu Heart Study, which only enrolled male individuals of Japanese ancestry, observed positive aCL IgG/IgM or aβ2GPI IgG among 12% of healthy male individuals.^20^ Our study identified aPL in 14.5% of individuals, with one-third of those positives at a moderate or high titer. To our knowledge, our study is the first to evaluate the presence of both criteria and noncriteria aPL by solid-phase assays in a diverse, healthy population in which longitudinal data regarding ASCVD events are available.

The impact of sex and race and ethnicity on the frequency of aPL has not been systematically investigated. Systemic autoimmune conditions are more often diagnosed in female individuals. For example, SLE is 10 times more common in females of reproductive age as compared with males.^38^ Given the close association between SLE and APS, it is possible that sex may play a role in the frequency of aPL. Here, we found that female participants were more likely to have aCL IgM and aPS/PT IgM; however, this difference disappeared when limiting the analysis to aPL present at moderate or high titer. SLE develops in Black female individuals at rates that are 3 to 4 times higher than their White counterparts.^39^ While it is sometimes suggested that Black patients with SLE are less likely to have criteria aPL,^33^ the data to support this assertion are not strong. Furthermore, enrollment in APS clinical trials often skews toward White patients.^40^ Here, we found that the prevalence of any positive aPL by solid-phase assays is comparable among Black, White, and Hispanic individuals. Robust efforts will be needed to ensure that all stakeholder groups are represented in future APS clinical trials.

In a European multicenter registry of 1000 patients with a clinical diagnosis of APS, ischemic stroke was reported in 19.8% of patients, whereas MI was the second most common cause of death during follow-up (5.5%).^41^ In clinical practice, most patients only have aPL assessed after a devastating thrombotic event. Utilizing selected aPL as potential biomarkers for risk stratifying seemingly healthy individuals for future ASCVD events has not been explored in the past. One notable finding of our study is that aCL IgA and aβ2GPI IgA, even at low titer and tested at a single time point, are independently associated with future ASCVD events. The role of some aPL by solid-phase assays as nontraditional markers of ASCVD risk warrants further research.

We found that aβ2GPI IgA associated with impaired CEC, the primary antiatherosclerotic function of HDL. To our knowledge, this is the first study to explore the association between aPL and CEC. One crucial pathogenic mechanism of IgG aPL is their ability to promote thromboinflammatory NET release,^22,42^ although this phenotype has not been defined for IgA isotypes. Reactive oxygen species released during NET formation can oxidize HDL and interfere with its function,^35^ while circulating NET remnants correlate with impaired CEC in patients with SLE.^43^ Here, we did not find an association between CEC and NET formation, suggesting that other mechanisms may be relevant. Antibodies against apolipoprotein A-1 (ApoA1), a central component of HDL, have been associated with elevated CVD risks among patients with rheumatoid arthritis.^2,44^ One recent study found higher levels of anti-HDL antibodies in patients with APS and prior arterial thrombosis.^45^ Future studies should assess the effect of various aPL species and isotypes on HDL function.

Vascular endothelial dysfunction has also been suggested to play an essential role in aPL-mediated CVD. aPL can activate endothelial cells through ApoER2, impairing nitric oxide production and causing a proadhesive and proinflammatory endothelial phenotype.^46^ These mechanisms—focused mainly on the IgG isotype of criteria aPL—have primarily been studied in the context of systemic autoimmune diseases. The potential role of noncriteria aPL in promoting CVD in a healthy population has not been studied. Here, we found that aβ2GPI IgA–positive plasma activated cultured coronary endothelial cells to express surface adhesion molecules integral to inflammation and thrombosis, namely E-selectin, ICAM-1, and VCAM-1. It is noteworthy that aβ2GPI IgG was only rarely detected in our cohort, and its role in endothelial cell activation was therefore not characterized here. Future mechanistic studies on aβ2GPI isotypes beyond IgG are warranted.

### Limitations

We recognize several important limitations to our study. Sufficient citrated plasma was not available for testing lupus anticoagulant; our results therefore do not comprehensively evaluate all relevant aPL. We also do not have serial samples taken 12 weeks apart to confirm the aPL persistence required for formal classification of APS^14^; as such, we cannot determine the prevalence of classifiable APS in the general population. Furthermore, we could not evaluate potential associations between circulating aPL and some well-recognized APS clinical manifestations, such as venous thrombosis and pregnancy morbidity, as DHS was designed to capture only ASCVD events. Acknowledging those limitations, we emphasize that the goal of our study was not to classify APS, but rather to ask whether the presence of some aPL by solid-phase assays at a single time point in the general population might associate with future ASCVD risk. In fact, using single time point aPL would seem likely to bias results toward the null hypothesis. Despite this, our study found that the presence of aCL IgA and aβ2GPI IgA, even at a single time point, were associated with future ASCVD events. Of course, it is essential to acknowledge that misclassification bias due to the single time point measurement could contribute to the lack of an observed association between other aPL subtypes and ASCVD events. Taken together, we recognize that all these associations should be viewed as hypothesis-generating and warrant confirmation. Additionally, we acknowledge that the relatively small number of ASCVD events limits the power to detect interactions related to race and ethnicity. Thus, more extensive studies, designed with serial blood sampling over approximately 3 months, would seem ideal to further investigate all aPL subtypes in the general population.

## Conclusions

This population-based cohort study found that the presence of some aPL assessed by solid-phase assays, even at a single time point, were independently associated with future ASCVD events. Further confirmatory studies are needed, including those that can help to determine the extent to which therapeutic strategies might mitigate aPL-associated risks.

## Notes

Supplement 1. **eMethods.** Supplemental Methods **eTable 1.** Sex Variation of Antiphospholipid Antibodies Based on Manufacturer’s Cut-off in Dallas Heart Study (n=2427) **eTable 2.** Sex Variation of Antiphospholipid Antibodies Based on Moderate- to High-Titer Cut-off in Dallas Heart Study (n=2427) **eTable 3.** Ethnic Variation of Antiphospholipid Antibodies in Dallas Heart Study (n=2427) **eTable 4.** Antiphospholipid Antibodies and Antinuclear Antibodies (ANA) **eTable 5.** Association of Incident Cardiovascular Events in Unadjusted Model and Model Adjusted for Known Cardiovascular Disease Risk Factors **eFigure 1.** Association of aPL Subtypes With Future Atherosclerotic Cardiovascular Disease (ASCVD) Events (95% Simultaneous Confidence Interval Reported) **eFigure 2.** Circulating NETs in Dallas Heart Study (DHS) Participants With Positive aβ2GPI IgA, as Compared With Gender, Age, Ethnicity Matched Controls **eFigure 3.** Schematic Illustration of In-Cell ELISA for Human Coronary Artery Endothelial Cells (HCAEC)
eReferences

Supplement 2.
Data Sharing Statement
