# Supplementary material for: Prevalence of Antiphospholipid Antibodies and Association With Incident Cardiovascular Events
Source: JAMA Netw Open. 2023 Apr 4;6(4):e236530. doi: 10.1001/jamanetworkopen.2023.6530 (PMC10074226; doi:10.1001/jamanetworkopen.2023.6530)
Supplement: Supplement 1. — eMethods. Supplemental Methods eTable 1. Sex Variation of Antiphospholipid Antibodies Based on Manufacturer’s Cut-off in Dallas Heart Study (n=2427) eTable 2. Sex Variation of Antiphospholipid Antibodies Based on Moderate- to High-Titer Cut-off in Dallas Heart Study (n=2427) eTable 3. Ethnic Variation of Antiphospholipid Antibodies in Dallas Heart Study (n=2427) eTable 4. Antiphospholipid Antibodies and Antinuclear Antibodies (ANA) eTable 5. Association of Incident Cardiovascular Events in Unadjusted Model and Model Adjusted for Known Cardiovascular Disease Risk Factors eFigure 1. Association of aPL Subtypes With Future Atherosclerotic Cardiovascular Disease (ASCVD) Events (95% Simultaneous Confidence Interval Reported) eFigure 2. Circulating NETs in Dallas Heart Study (DHS) Participants With Positive aβ2GPI IgA, as Compared With Gender, Age, Ethnicity Matched Controls eFigure 3. Schematic Illustration of In-Cell ELISA for Human Coronary Artery Endothelial Cells (HCAEC) eReferences [file jamanetwopen-e236530-s001.pdf]

Supplemental Online Content

Zuo Y, Navaz S, Liang W, et al. Prevalence of antiphospholipid antibodies and association with incident cardiovascular events. *JAMA Netw Open.* 2023;6(4):e236530. doi:10.1001/jamanetworkopen.2023.6530

eMethods. Supplemental Methods

eTable 1. Sex Variation of Antiphospholipid Antibodies Based on Manufacturer's Cut-off in Dallas Heart Study (n=2427)

eTable 2. Sex Variation of Antiphospholipid Antibodies Based on Moderate- to High-Titer Cut-off in Dallas Heart Study (n=2427)

eTable 3. Ethnic Variation of Antiphospholipid Antibodies in Dallas Heart Study (n=2427)

eTable 4. Antiphospholipid Antibodies and Antinuclear Antibodies (ANA)

eTable 5. Association of Incident Cardiovascular Events in Unadjusted Model and Model Adjusted for Known Cardiovascular Disease Risk Factors

eFigure 1. Association of aPL Subtypes With Future Atherosclerotic Cardiovascular Disease (ASCVD) Events (95% Simultaneous Confidence Interval Reported)

eFigure 2. Circulating NETs in Dallas Heart Study (DHS) Participants With Positive aβ2GPI IgA, as Compared With Gender, Age, Ethnicity Matched Controls

eFigure 3. Schematic Illustration of In-Cell ELISA for Human Coronary Artery Endothelial Cells (HCAEC)

eReferences

This supplemental material has been provided by the authors to give readers additional information about their work.

© 2023 Zuo Y et al. *JAMA Network Open.*

eMethods. Supplemental Methods

Study population. The Dallas Heart Study (**DHS**) is a multi-phased, prospective cohort study designed to produce unbiased population estimates of biologic variables that influence cardiovascular health at the community level and support hypothesis-driven research on the mechanisms underlying these differences. A probability-based population cohort of Dallas County including English- or Spanish-speaking adults aged 18 to 65 years was surveyed and enrolled in 1999 during Dallas Heart Study phase 1 (**DHS1**)[1]. To address the under-representation of Black individuals in longitudinal cohort studies, oversampling of Black individuals was performed so that Black participants would represent 50% of the cohort. DHS phase 2 (**DHS2**) was conducted between September 2007 and December 2009 as a comprehensive follow-up study of participants who completed DHS1. At each phase, participants underwent extensive phenotyping including multicomponent cardiovascular, body composition, and brain imaging (phase 2 only), as well as biospecimen collection for genomics and blood biomarkers. Cardiovascular risk factors were assessed at the time of both the DHS1 and DHS2 visits, as previously described[1]. Coronary artery calcium (**CAC**) was assessed by electron-beam CT and CAC score was determined with the Agatston method[2].

Quantification of aPL. aPL were quantified from thawed frozen plasma using Quanta Lite® ACA IgG, ACA IgM, ACA IgA, β2GPI IgG, β2GPI IgM, β2GPI IgA, aPS/PT IgG, and aPS/PT IgM kits (Werfen North America) according to the manufacturer's instructions as described previously[3]. All assays are approved for clinical use and received 510(k) clearance from the FDA. Positive aPL were defined as results equal to or above the manufacturer's defined

© 2023 Zuo Y et al. *JAMA Network Open.*

threshold value. Moderate- or high-titer aPL were defined as ≥40 corresponding units (GPL/MPL/APL for aCL IgG/M/A; SGU/SMU/SAU for aβ2GPI IgG/M/A; and arbitrary units for aPS/PT IgG/M)[4]. All measurements were performed by investigators blinded to clinical outcomes or other DHS data.

Cholesterol Efflux Capacity (CEC). CEC was assessed in stored blood specimens from DHS2 participants via a CEC assay that measures the efflux of radiolabeled cholesterol from J774 macrophages in the presence of apolipoprotein B (**ApoB**)-depleted plasma. Individual efflux values were normalized to values obtained with a pool of 2% ApoB-depleted plasma from selected controls thus making the efflux values unitless. The detailed methodology has been described previously[5, 6].

Quantification of myeloperoxidase-DNA complexes. Myeloperoxidase-DNA complexes were quantified among those participants similarly to what has been previously described[7]. This protocol used several reagents from the Cell Death Detection ELISA kit (Roche Diagnostics, Indianapolis, IN). First, a high-binding 96-well plate (Costar by Fisher Scientific) was coated overnight at 4ºC with anti-human myeloperoxidase antibody (Bio-Rad 0400-0002), diluted to a concentration of 1 µg/ml in coating buffer (Cell Death kit by Sigma). The plate was washed 2 times with wash buffer (0.05% Tween 20 in PBS), and then blocked with 4% bovine serum albumin in PBS (supplemented with 0.05% Tween 20) for 2 hours at room temperature. The plate was again washed 5 times, before incubating for 90 minutes at room temperature with 10% serum or plasma in the aforementioned blocking buffer (without Tween 20). The plate was washed 5 times, and then incubated for 90 minutes at room temperature with 10x anti-DNA antibody (HRP-conjugated; from the Cell Death kit) diluted 1:100 in blocking buffer. After 5 more washes, the plate was developed with 3,3',5,5'-Tetramethylbenzidine (**TMB**) substrate (Invitrogen) followed by a 2N sulfuric acid stop solution. Absorbance was measured at a

© 2023 Zuo Y et al. *JAMA Network Open.*

wavelength of 450 nm using a Cytation 5 Cell Imaging Multi-Mode Reader (BioTek). Data were normalized to *in vitro*-prepared NET standards included on every plate, which were quantified based on their DNA content.

In-cell ELISA. Human coronary artery endothelial cell (**HCAEC**) activation was assessed by an in-cell ELISA, which measured the surface expression of E-selectin, ICAM-1, and VCAM-1 on endothelial cells[8]. Briefly, monolayers of HCAEC in 96-well microplates were incubated with 2.5% DHS2 plasma (5 from participants with isolated high titer aβ2GPI IgA and 6 from participants without any aPL) for 6 hours and then fixed using the same volume of 4% paraformaldehyde for 15 minutes. Cells were blocked with 2x blocking solution (catalog 8209728, Abcam) at room temperature for 2 hours. They were then incubated with 5 µg/ml primary mouse anti-human antibodies against E-selectin (catalog BBA26, R&D), VCAM-1 (catalog BBA5, R&D), or ICAM-1 (ab2213, Abcam) at 4°C overnight. Next, 100 µl of diluted horseradish peroxidase-conjugated rabbit anti-mouse IgG (1:2000, Jackson ImmunoResearch) in 1x blocking solution was added and incubated at room temperature for 2 hours. After washing thoroughly, the plate was developed with TMB substrate, and the absorbance was then measured at a wavelength of 650 nm using a Cytation 5 Cell Imaging Multi-Mode Reader (BioTek). The signals were corrected by subtracting the mean signal of wells incubated in the absence of the primary antibody from all other readings.

© 2023 Zuo Y et al. *JAMA Network Open.*

**eTable 1:** Sex Variation of Antiphospholipid Antibodies Based on Manufacturer's Cut-off in Dallas Heart Study (n=2427)			
	**Male** (n=1028, 42.4%)	**Female** (n=1399, 57.6%)	
**aPL**	**Number positive (manufacturer's threshold)**		Adjusted P value*
aCL IgG	12 (1.2%)	14 (1%)	0.89
aCL IgM	*50 (4.9%)*	*106 (7.6%)*	***0.035***
aCL IgA	4 (0.4%)	7 (0.5%)	0.89
aβ2GPI IgG	10 (1.0%)	11 (0.8%)	0.89
aβ2GPI IgM	26 (2.5%)	37 (2.6%)	0.9
aβ2GPI IgA	25 (2.4%)	37 (2.6%)	0.89
aPS/PT IgG	6 (0.6%)	12 (0.9%)	0.89
aPS/PT IgM	*26 (2.5%)*	*56 (4.0%)*	***0.035***
Any positive	*131 (12.7%)*	*222 (15.9%)*	0.1
Three positive aPL	4 (0.4%)	13 (0.9%)	0.35
*adjusted for multiple comparisons by modified Benjamini-Hochberg Step-up Method			

© 2023 Zuo Y et al. *JAMA Network Open.*

**eTable 2:** Sex Variation of Antiphospholipid Antibodies Based on Moderate- to High-Titer Cut-off in Dallas Heart Study (n=2427)			
	**Male** (n=1028, 42.4%)	**Female** (n=1399, 57.6%)	
**aPL**	Number positive (≥40 U cut-off)		Adjusted P value*
aCL IgG	2 (0.2%)	5 (0.4%)	0.94
aCL IgM	12 (1.2%)	24 (1.7%)	0.76
aCL IgA	2 (0.2%)	4 (0.4%)	1
aβ_2GPI IgG	5 (0.5%)	5 (0.4%)	0.94
aβ_2GPI IgM	11 (1.0%)	15 (1.1%)	1
aβ_2GPI IgA	9 (0.9%)	20 (1.4%)	0.76
aPS/PT IgG	3 (0.3%)	8 (0.6%)	0.76
aPS/PT IgM	17 (1.7%)	31 (2.2%)	0.76
Any positive	55 (5.4%)	98 (7%)	0.76
Three positive aPL	0	2 (0.1%)	0.85
*adjusted for multiple comparisons by modified Benjamini-Hochberg Step-up Method			

© 2023 Zuo Y et al. *JAMA Network Open.*

**eTable 3:** Ethnic variation of antiphospholipid antibodies in Dallas Heart Study (n=2427)					
	**Black (n=1244)**	**Hispanic (n=339)**	**White (n=796)**	**Other* (n=48)**	**Adjusted P value‡**
**aPL**	Number positive and % (≥40 U cut-off)				
aCL IgG	5 (0.4%)	0	2 (0.3%)	0	0.76
aCL IgM	23 (1.9%)	2 (0.6%)	10 (1.3%)	1 (2.0%)	0.76
aCL IgA	5 (0.4%)	0	1 (0.1%)	0	0.76
aβ_2GPI IgG	6 (0.5%)	1 (0.3%)	3 (0.4%)	0	0.9
aβ_2GPI IgM	12 (1.0%)	3 (0.9%)	11 (1.4%)	0	0.76
aβ_2GPI IgA	*18 (1.5%)*	*10 (3.0%)*	*1 (0.1%)*	*0*	***0.005***
aPS/PT IgG	5 (0.4%)	3 (0.9%)	3 (0.4%)	0	0.76
aPS/PT IgM	24 (1.9%)	9 (2.7%)	15 (1.9%)	0	0.76
Any positive	87 (7%)	25 (7.4%)	40 (5%)	1 (2.0%)	0.75
Three positive aPL	1 (0.08%)	1 (0.3%)	0	0	0.76

*****included Asian or Pacific islander, Native American, and other self-reported race/ethnicity by Dallas Heart Study participants
‡adjusted for multiple comparison by modified Benjamini-Hochberg Step-up Method

© 2023 Zuo Y et al. *JAMA Network Open.*

**eTable 4:** Antiphospholipid antibodies and antinuclear antibodies (**ANA**)						
	**ANA positive** (n=103)	**ANA negative** (n=2052)	**Chi square P value***	**ANA positive** (n=103)	**ANA negative** (n=2052)	**Chi square P value***
**aPL**	Number positive (%) (manufacturer's threshold)			Number positive (%) (titer ≥40 U)		
aCL IgG	1 (1.0%)	23 (1.1%)	0.89	0	7 (0.3%)	n/a
aCL IgM	9 (8.7%)	137 (6.7%)	0.75	3 (2.9%)	28 (1.4%)	0.24
aCL IgA	0	8 (0.4%)	0.89	0	3 (0.2%)	n/a
aβ2GPI IgG	1 (1.0%)	17 (1.0%)	0.89	0	9 (0.4%)	n/a
aβ2GPI IgM	2 (2.0%)	57 (2.8%)	0.76	2 (1.9%)	22 (1.0%)	0.41
aβ2GPI IgA	4 (3.9%)	53 (2.6%)	***0.0009***	*4 (3.9%)*	*22 (1.0%)*	***0.02***
aPS/PT IgG	*4 (3.9%)*	*12 (0.6%)*	0.14	*3 (2.9%)*	*7 (0.3%)*	**0.0012**
aPS/PT IgM	*7 (6.8%)*	*62 (3.0%)*	0.3	*5 (4.9%)*	*36 (1.8%)*	***0.03***
Any positive	21 (20%)	297 (14%)	0.89	*15 (15%)*	*120 (5.9%)*	***0.0012***
Three positive aPL	1 (1.0%)	15 (0.7%)	0.89	0	2 (0.1%)	N/A
ANA-positive threshold ≥65 EU, equivalent to ≥ 1:160. ANA were measured at DHS1 and aPL at DHS2. *adjusted for multiple comparisons by modified Benjamini-Hochberg Step-up Method						

© 2023 Zuo Y et al. *JAMA Network Open.*

**eTable 5:** Association of incident cardiovascular events in unadjusted model and model adjusted for known cardiovascular disease risk factors.						
	ASCVD in aPL-positive group: N with events (K-M estimated event rate)	ASCVD in aPL-negative group" N with events (K-M estimated event rate)	Unadjusted HR (95% CI)	P value*	Adjusted HR (95% CI)	P value*
+ aCL IgG (≥20)	1 (4.35%)	124 (5.35%)	0.81 (0.11-5.81)	0.836	0.83 (0.12-5.95)	0.915
+ aCL IgG (≥40)	0 (0%)	125 (5.35%)	NA		NA	
+ aCL IgM (≥20)	4 (2.70%)	121 (5.52%)	0.49 (0.18-1.34)	0.306	0.68 (0.25-1.86)	0.738
+ aCL IgM (≥40)	1 (3.13%)	124 (5.37%)	0.60 (0.08-4.30)	0.797	0.64 (0.09-4.61)	0.826
+ aCL IgA (≥20)	3 (30%)	122 (5.23%)	*6.25 (1.99-19.66)*	***0.007***	*4.92 (1.52-15.98)*	***0.026***
+ aCL IgA (≥40)	3 (50%)	122 (5.22%)	*10.72 (3.41-33.72)*	***0.001***	*9.01 (2.73-29.72)*	***0.004***
+ aβ2GPI IgG (≥20)	0 (0%)	125 (5.38%)	NA		NA	
+ aβ2GPI IgG (≥40)	0 (0%)	125 (5.36%)	NA		NA	
+ aβ2GPI IgM (≥20)	4 (6.56%)	121 (5.31%)	1.31 (0.48-3.54)	0.797	1.36 (0.50-3.71)	0.799
+ aβ2GPI IgM (≥40)	3 (11.54%)	122 (5.27%)	2.34 (0.75-7.37)	0.306	2.46 (0.77-7.81)	0.277
+ aβ2GPI IgA (≥20)	9 (15.52%)	116 (5.08%)	*3.31 (1.68-6.52)*	***0.003***	*2.91 (1.32-6.41)*	***0.026***
+ aβ2GPI IgA (≥40)	5 (18.52%)	120 (5.19%)	*4.03 (1.65-9.86)*	***0.008***	*4.09 (1.45-11.54)*	***0.026***
+ aPS/PT IgG (≥30)	0 (0%)	125 (5.38%)	NA		NA	
+ aPS/PT IgG (≥40)	0 (0%)	125 (5.36%)	NA		NA	
+ aPS/PT IgM (≥30)	5 (6.17%)	120 (5.31%)	1.20 (0.49-2.92)	0.797	0.95 (0.398-2.36)	0.915
+ aPS/PT IgM (≥40)	3 (6.38%)	122 (5.32%)	1.22 (0.39-3.83)	0.797	0.80 (0.25-2.54)	0.826
Any + aPL‡	22 (6.53%)	103 (5.14%)	1.30 (0.82-2.07)	0.421	1.28 (0.79-2.09)	0.594
Any + aPL (≥40)	14 (9.72%)	111 (5.05%)	*2.03 (1.16-3.53)*	***0.033***	1.74 (0.97-3.12)	0.169

ASCVD=atherosclerotic cardiovascular disease; N=number; K-M=Kaplan-Meier; HR=hazard ratio; CI=confidence interval. NA= HR not calculable due to 0 events in aPL group. ‡≥30 cut-off for aPS/PT IgG/IgM and ≥20 cut-off for the rest of antiphospholipid antibodies. *adjusted for multiple comparisons by modified Benjamini-Hochberg Step-up Method

© 2023 Zuo Y et al. *JAMA Network Open.*

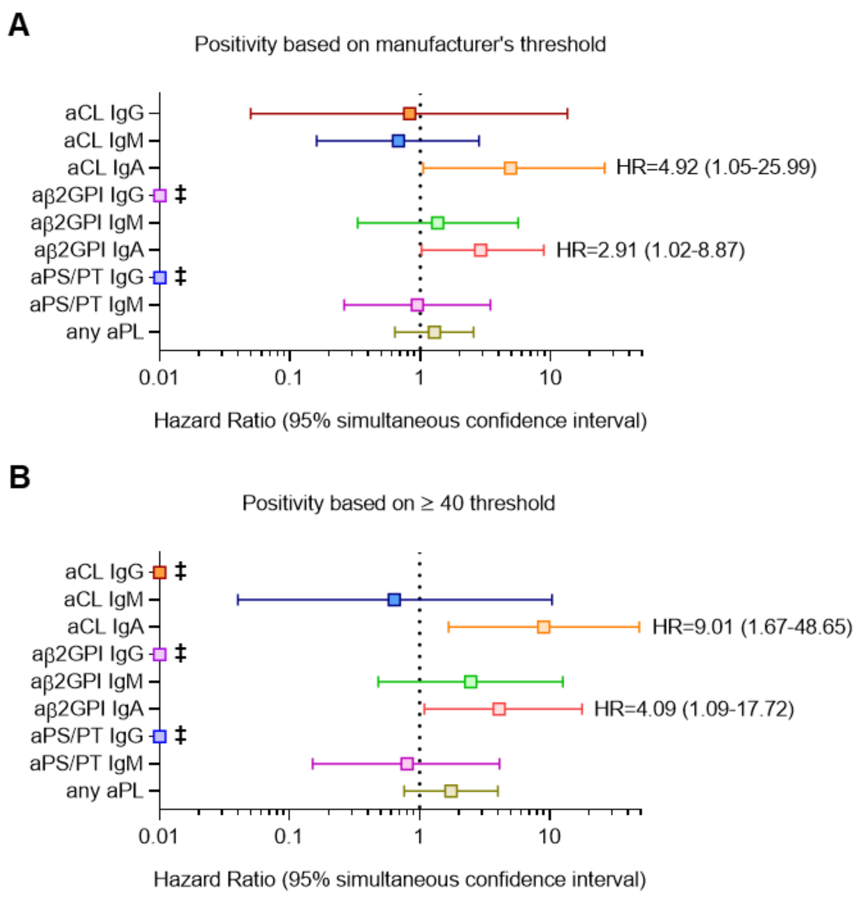

eFigure 1: Association of aPL subtypes with future atherosclerotic cardiovascular disease (ASCVD) events (95% simultaneous confidence interval reported). Association between positive aPL based on either manufacturer's threshold (**A**) or ≥40 U threshold (**B**) and future ASCVD events were assessed in Cox proportional hazard models adjusted for known cardiovascular risks including age, ethnicity, smoking history, hypertension, diabetes, and lipid profiles. To ascertain our findings and improve coverage probability, here we reported 95% simultaneous confidence intervals. HR=Hazard ratio; **‡** HR not calculable due to 0 events in aPL group.

© 2023 Zuo Y et al. *JAMA Network Open.*

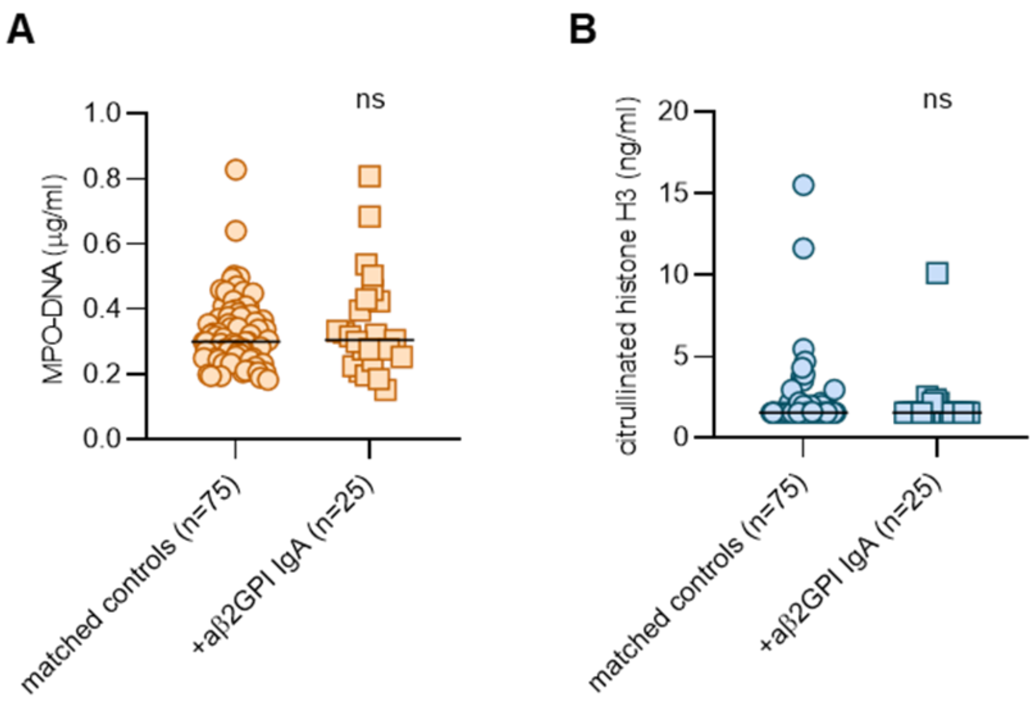

eFigure 2: Circulating NETs in Dallas Heart Study (DHS) participants with positive aβ2GPI IgA, as compared with gender, age, ethnicity matched controls. Plasma was tested for myeloperoxidase-DNA complexes (MPO-DNA) (**A**), and citrullinated histone H3 (**B**). N=75 for the control group and n=25 for the aβ2GPI IgA-positive group. Comparisons were by Mann-Whitney test, ns=nonsignificant.

© 2023 Zuo Y et al. *JAMA Network Open.*

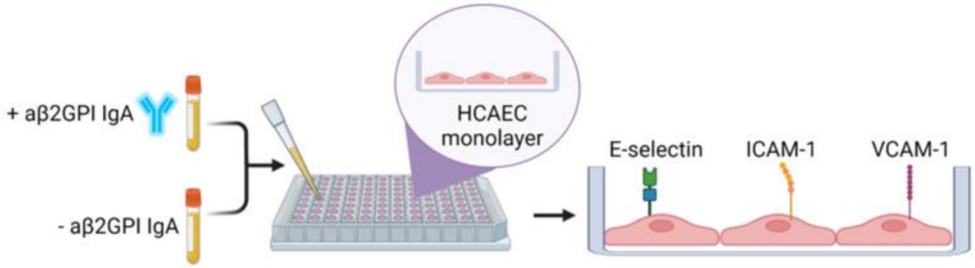

eFigure 3: Schematic illustration of in-cell ELISA for human coronary artery endothelial cells (HCAEC). HCAEC were cultured for 6 hours with plasma from either aβ2GPI-positive or -negative DHS2 participants. Cells were then fixed, and surface expression of E-selectin, ICAM-1, or VCAM-1 was quantified.

© 2023 Zuo Y et al. *JAMA Network Open.*

eReferences

1. Victor RG, Haley RW, Willett DL, et al. The Dallas Heart Study: a population-based probability sample for the multidisciplinary study of ethnic differences in cardiovascular health. *Am J Cardiol*. Jun 15 2004;93(12):1473-80. doi:10.1016/j.amjcard.2004.02.058
2. Paixao AR, Berry JD, Neeland IJ, et al. Coronary artery calcification and family history of myocardial infarction in the Dallas heart study. *JACC Cardiovasc Imaging*. Jul 2014;7(7):679-86. doi:10.1016/j.jcmg.2014.04.004
3. Zuo Y, Estes SK, Ali RA, et al. Prothrombotic autoantibodies in serum from patients hospitalized with COVID-19. *Sci Transl Med*. Nov 18 2020;12(570)doi:10.1126/scitranslmed.abd3876
4. Miyakis S, Lockshin MD, Atsumi T, et al. International consensus statement on an update of the classification criteria for definite antiphospholipid syndrome (APS). *J Thromb Haemost*. Feb 2006;4(2):295-306. doi:10.1111/j.1538-7836.2006.01753.x
5. Akinmolayemi O, Saldanha S, Joshi PH, et al. Cholesterol efflux capacity and its association with prevalent metabolic syndrome in a multi-ethnic population (Dallas Heart Study). *PLoS One*. 2021;16(9):e0257574. doi:10.1371/journal.pone.0257574
6. Sankaranarayanan S, Kellner-Weibel G, de la Llera-Moya M, et al. A sensitive assay for ABCA1-mediated cholesterol efflux using BODIPY-cholesterol. *J Lipid Res*. Dec 2011;52(12):2332-2340. doi:10.1194/jlr.D018051
7. Zuo Y, Yalavarthi S, Shi H, et al. Neutrophil extracellular traps in COVID-19. *JCI Insight*. Apr 24 2020;doi:10.1172/jci.insight.138999
8. Shi H, Zuo Y, Navaz S, et al. Endothelial cell-activating antibodies in COVID-19. *Arthritis Rheumatol*. Feb 17 2022;doi:10.1002/art.42094

© 2023 Zuo Y et al. *JAMA Network Open.*
