# Supplementary material for: Prevalence of Antiphospholipid Antibodies and Association With Incident Cardiovascular Events
Source: JAMA Netw Open. 2023 Apr 4;6(4):e236530. doi: 10.1001/jamanetworkopen.2023.6530 (PMC10074226; doi:10.1001/jamanetworkopen.2023.6530)
Supplement: Supplement 2. — Data Sharing Statement [file jamanetwopen-e236530-s002.pdf]

Data Sharing Statement

Zuo. Prevalence of Antiphospholipid Antibodies and Association With Incident Cardiovascular Events. *JAMA Netw Open*. Published April 04, 2023. doi:10.1001/jamanetworkopen.2023.6530

Data
Data available: No
